# Effects of the switch from dulaglutide to tirzepatide on glycemic control, body weight, and fatty liver: a retrospective study

**DOI:** 10.1007/s40200-024-01472-w

**Published:** 2024-07-26

**Authors:** Toshitaka Sawamura, Ren Mizoguchi, Ai Ohmori, Mitsuhiro Kometani, Takashi Yoneda, Shigehiro Karashima

**Affiliations:** 1 Division Department of Internal Medicine, Asanogawa General Hospital, 83 Kosakamachi, Kanazawa, Ishikawa 920-8621 Japan; 2 Department of Health Promotion and Medicine of the Future, Kanazawa University, 13-1 Takaramachi, Kanazawa, 920-8641 Japan

**Keywords:** Type 2 diabetes, Tirzepatide, Dulaglutide, GLP-1, GIP, NAFLD

## Abstract

**Objectives:**

Tirzepatide belongs to a new class of anti-diabetic agents that stimulate both glucagon-like peptide-1 and glucose-dependent insulinotropic polypeptide receptors, resulting in a greater blood glucose-lowering effect and body weight reduction than glucagon-like peptide-1 analogs. However, data on the effects of switching from glucagon-like peptide-1 analogs to tirzepatide on the blood glucose level, body weight, and liver functions are unavailable.

**Methods:**

Data from 40 patients with type 2 diabetes who received a prescription change from dulaglutide to tirzepatide were retrospectively analyzed at the 3 and 6 months after the switch. The analyzed data included glycosylated hemoglobin, body weight, aspartate aminotransferase, alanine aminotransferase, γ-glutamyl transpeptidase levels, and fibrosis-4 index.

**Results:**

Six months after the treatment switch, average reductions of 1.2% and 3.6 kg were observed in the glycosylated hemoglobin and body weight, respectively. The change in glycosylated hemoglobin level was negatively correlated with the baseline glycosylated hemoglobin level. However, body weight reduction was observed regardless of the baseline characteristics. Moreover, the aspartate aminotransferase, alanine aminotransferase, and γ-glutamyl transpeptidase levels decreased 6 months after the switch. Reductions in alanine aminotransferase levels was greater in patients with higher baseline aspartate aminotransferase, alanine aminotransferase, and γ-glutamyl transpeptidase levels. Although the fibrosis-4 index did not improve during the study period, a trend toward a decrease was observed in patients with a higher baseline fibrosis-4 index.

**Conclusions:**

Switching from dulaglutide to tirzepatide has a beneficial effect on the blood glucose level, body weight, and liver function in patients with type 2 diabetes.

## Introduction

Type 2 diabetes is characterized by hyperglycemia due to both pancreatic beta-cell dysfunction and insulin resistance. Elevated blood glucose levels lead to diabetes-related complications and mortality [1]. Therefore, patients with diabetes require some therapeutic interventions for the control of blood glucose levels. The pharmacologic approach for the treatment of type 2 diabetes by the American Diabetes Association (ADA) recommends treatment selection based on metformin, focusing on body weight (BW), hypoglycemia avoidance, and additional effects for coexisting disease [2]. However, type 2 diabetes in East Asians is characterized primarily by beta-cell dysfunction, and with less adiposity and less insulin resistance compared with that in Westerners [3]. Thus, the Japanese treatment strategy should differ from that for Westerners. For example, Dipeptidyl peptidase-4 (DPP-4) inhibitors are the most common prescription in real-world clinical practice [4] and have strong blood glucose-lowering effects in the Asian population [5], despite not being strongly recommended in the strategy by ADA [2]. Genome-wide association studies have shown that three variants have distinct minor allele frequency spectra between people of Japanese and European ancestry, including variants of genes related to pancreatic acinar cells and Glucagon-like peptide-1 (GLP-1)-induced insulin secretion [6]. Therefore, stimulation of the GLP-1 receptor is likely to enhance insulin secretion strongly in Japanese patients with diabetes, and incretin enhancers are beneficial for treating Japanese patients with diabetes.

However, it has recently been shown that attention should be paid not only to beta-cell dysfunction but also to insulin sensitivity in Japanese patients with type 2 diabetes. In East Asian populations, visceral fat accumulates more easily than subcutaneous fat in Westerners [7]. In recent years, BW, and consequently the body mass index (BMI), increased in Japanese patients with type 2 diabetes [8]. Fat accumulation in the liver is associated with not only hepatic insulin resistance, but also with systemic insulin resistance through skeletal muscle insulin resistance [9]. Thus, the BW gain observed in Japanese in recent years is associated with visceral fat accumulation and insulin resistance [10]. Improving insulin resistance by avoiding visceral fat accumulation is one of the important tasks.

GLP-1 analogs are agents that stimulate mainly postprandial insulin secretion and slow gastric emptying, resulting in decreasing blood glucose levels. Furthermore, the effect of the decrease in appetite is reported [11]. Therefore, this class of agents is positioned as strong blood glucose-lowering agents combined with reducing BW in the pharmacologic approach by ADA [2]. Furthermore, this class of agents also improves liver injury in patients with fatty liver and diabetes [12]. However, in practice, some patients have poor glycemic control, require further weight loss and improvement of liver function, despite using GLP-1 analogs.

Tirzepatide is a novel anti-diabetic agent that acts as a dual glucose-dependent insulinotropic polypeptide (GIP) and GLP-1 receptor agonist. Its structure is primarily based on the glucose-dependent insulinotropic polypeptide amino acid sequence and includes a C20 fatty diacid moiety [13]. Its half-life of approximately 5 days allows for once-weekly subcutaneous injection [13]. GIP induces glucose-dependent insulin secretion and decreases blood glucose levels were observed in research on rat [14] and humans [15]. However, the glucose-dependent insulinotropic effects is induced not only by GIP but also by GLP-1 in humans. Between these two hormones, postprandial insulin secretion induced by GIP is approximately twofold higher compared to that induced by GLP-1 in healthy human [16]. However, patients with type 2 diabetes often exhibit resistance to GIP, resulting in diminished GIP-mediated insulinotropic effects [17]. In research on rats, it has been reported that exposure to high blood glucose levels leads to reduced expression of GIP receptor messenger ribonucleic acid in pancreatic islets [18]. Consequently, the glucose-dependent insulinotropic effects by GIP might not function adequately in patients with diabetes. In addition to stimulating insulin secretion, pharmacological doses of GIP have been reported to decrease appetite and reduce BW in mice and these effects is canceled by a knockout of central GIP receptors [19]. These results suggests that GIP-induced decreasing appetite and BW are mediated through central GIP receptor signaling. In a clinical study, tirzepatide had stronger blood glucose-lowering and BW-decreasing effects than dulaglutide, which is a widely used GLP-1 analog in Japan [20]. Thus, switching from a GLP-1 analog to tirzepatide might be beneficial in patients who need greater blood glucose levels or BW reduction, despite using GLP-1 analogs. However, clinical data regarding the switching from GLP-1 analogs to tirzepatide are lacking. Therefore, this study evaluated the effects of the switching from dulaglutide to tirzepatide on blood glucose level, BW, and liver function.

## Material and methods

### Patient selection

This retrospective study was conducted at Asanogawa General Hospital and related hospitals of Asanogawa Medical Corporation (Kanazawa, Japan). Data of adult Japanese patients 18 years of age or older who received a prescription change from dulaglutide to tirzepatide between April 2023 and July 2023 were retrospectively analyzed. Patients with severe hyperglycemia (glycosylate hemoglobin [HbA1c] level > 10.0%), type 1 diabetes, pancreatic diabetes, pregnancy, liver cirrhosis, and severe renal dysfunction (estimated glomerular filtration rate [eGFR] < 30 mL/min/1.73m^2^), were excluded. Furthermore, patients with poor adherence to tirzepatide below 90% based on medical records and patients whose anti-diabetic medications were changed during the observation period were excluded. However, the adjustment of insulin dosage in patients with insulin therapy was allowed.

### Clinical records

Physical, laboratory, and medication data were acquired before and 3 ± 1 months, and 6 ± 1 months after the switch from dulaglutide to tirzepatide. Physical data included height, weight, and calculated BMI. Medication data included the dosages of tirzepatide and insulin, and the types of oral anti-diabetic agents. Laboratory data included the fasting plasma glucose (FPG), HbA1c, aspartate aminotransferase (AST), alanine aminotransferase (ALT), γ-glutamyl transpeptidase (γGTP), and creatinine (Cr) levels; Fibrosis 4 (FIB-4) index; eGFR; and C-peptide index (CPI). The FIB-4 index was calculated using age, AST, ALT, and the platelet count. CPI was evaluated based on fasting C-peptide immunoreactivity and FPG. Furthermore, evaluations of adverse effects were conducted based on the information documented in the medical records.

### Evaluation

During the study period, a total of 46 patients with type 2 diabetes received a prescription change from dulaglutide to tirzepatide. One patient discontinued tirzepatide within 6 months because of severe nausea. Another patient was excluded from the analysis because of severe renal dysfunction. Four patients changed their oral anti-diabetic agents for 6 months and were excluded from the analysis. Finally, 40 patients were included in the analysis.

The primary endpoint was the change in the HbA1c level at 6 ± 1 months after switching from dulaglutide to tirzepatide. The secondary endpoints were the change in HbA1c at 3 ± 1 months and the change in following items at 3 ± 1 and 6 ± 1 months: BW; BMI; AST, ALT, and γGTP levels; FIB-4 index. Additionally, we evaluated the factors affecting the changes in the HbA1c level, BW, ALT level, and FIB-4 index at 6 months.

### Ethics approval

This retrospective study was approved by the Ethics Committee of the Asanogawa Medical Corporation (No.243), and participants were recruited by the opt-out method. All procedures were performed in accordance with the 1964 Declaration of Helsinki and its later amendments.

#### Statistical analysis

Data are expressed as mean ± standard deviation (SD) unless otherwise noted. Data analyses were performed using the statistical software package EZR, version 1.55 (Saitama Medical Center, Jichi Medical University, Saitama, Japan), a graphical interface for R (The R Foundation for Statistical Computing, Vienna, Austria) [21], and R-studio, version 4.3.0. (RStudio, Boston, MA, United States). Variables were compared using a pairwise t-test or Wilcoxon signed-rank test. Significance was set at *p* < 0.05. The Bonferroni correction method was used for multiple comparisons. Associations between each parameter and the changes in the HbA1c level, BW, and liver parameters were examined using Pearson’s correlation coefficient analysis. Statistical significance was set at *p* < 0.05. No statistical sample size calculations were performed because this was a retrospective study.

## Results

The clinical characteristics of the 40 patients are summarized in Table 1. The mean age and duration of diabetes were 65.5 ± 14.5 years (33 to 91 years), and 13.5 ± 10.2 years (2 to 43 years), respectively, 32.5% of participants were female. At the time of tirzepatide initiation, the mean level of HbA1c levels and BMI were 7.9 ± 1.1%, and 27.8 ± 6.2 kg/m^2^, respectively. The mean number of anti-diabetes medications was 2.1 ± 0.9 items, and 45% (18/40) of patients used insulin. Sodium-glucose cotransporter 2 inhibitors, biguanides, glinide, sulfonylureas, α-glucosidase inhibitors, and thiazolidine were administrated to 73% (29/40), 48% (19/40), 18% (7/40), 14% (5/40), 10% (4/40), and 5% (2/40) patients, respectively. All the patients were switched from dulaglutide 0.75 mg/week to tirzepatide 2.5 mg/week. Tirzepatide dosages were adjusted according to the attached document. The maximum dose of tirzepatide in this study was 5 mg/week, because only 2.5 mg and 5 mg formulations were available in Japan at the time of study initiation. The mean dosage of tirzepatide at 3 months and 6 months were 4.8 ± 0.7 mg/week, and 4.7 ± 0.8 mg/week, respectively.

**Table 1:** Baseline characteristics of patients who received a prescription change from dulaglutide to tirzepatide

Baseline clinical characteristics	Value
Sex (male/female)	25/15
Age (years)	65.5 ± 14.5
Diabetes duration (years)	13.5 ± 10.2
BW (kg)	74.9 ± 18.3
BMI (kg/m^2^)	27.8 ± 6.2
HbA1c (%)	7.9 ± 1.1
Cr (mg/dL)	0.99 ± 0.76
eGFR (ml/min/1.73m^2^)	69.2 ± 28.6
AST (U/L)	30.6 ± 21.3
ALT (U/L)	34.6 ± 28.0
γ-GTP (mg/dL)	55.4 ± 71.2
FIB-4 index	1.49 ± 0.76
Fasting CPI	1.46 ± 0.52
Anti-diabetic treatment	
SGLT-2i/BG/Glinide/SU/α-GI/Tz/Insulin	29/19/7/5/4/2/18

Data are shown as the mean ± standard deviation

**Fig. 1:**
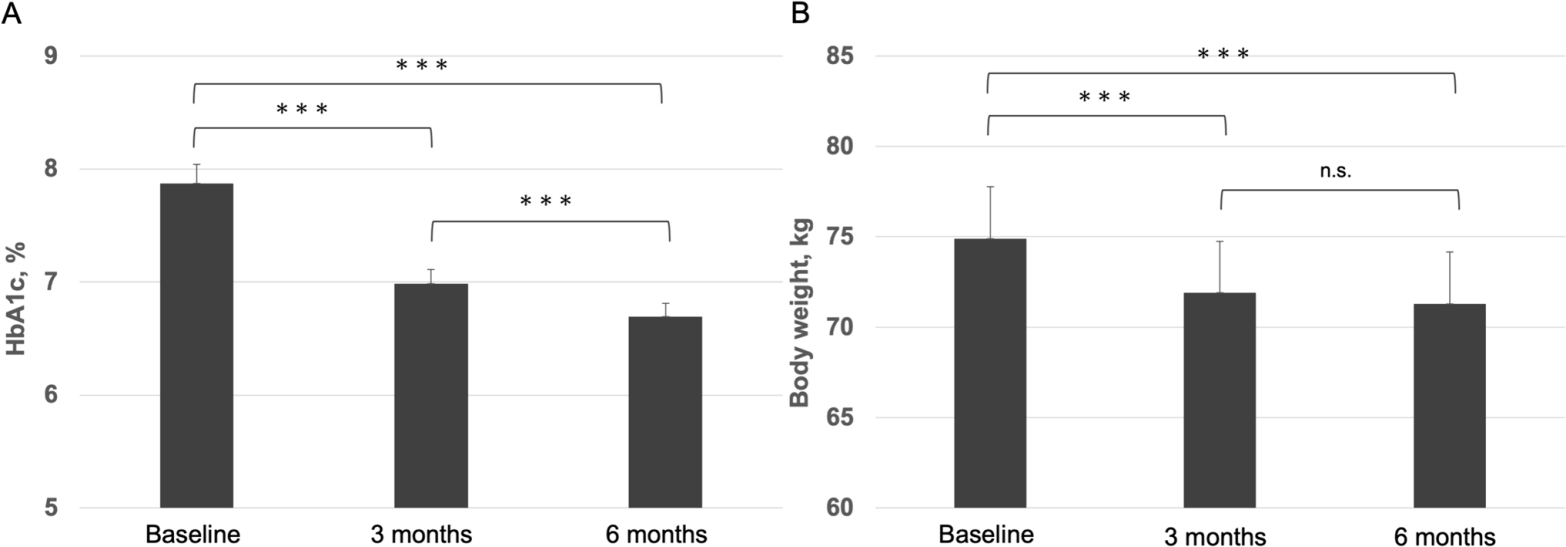
Changes in glycosylated hemoglobin levels (**A**) and body weight (**B**) 6 months after the switching from dulaglutide to tirzepatide. The *P*-values in this Figure are adjusted by the Bonferroni correction method. Data are shown as the mean ± standard error of the mean. ^***^ *P* < 0.001

As shown in Fig. 1A, the mean HbA1c levels decreased from 7.9 ± 1.1% at baseline to 7.0 ± 0.8% at 3 months (*p* < 0.001) and 6.7 ± 0.7% at 6 months (*p* < 0.001). The change in HbA1c levels from 3 to 6 months was also significant (*p* < 0.001). The mean BW decreased 74.9 ± 18.3 kg at baseline to 71.9 ± 17.9 kg at 3 months (*p* < 0.001) and 71.3 ± 18.0 kg at 6 months (*p* < 0.001). Although BW reduction was observed between 3 and 6 months, the difference was not significant (Fig. 1B). Changes in the AST, ALT, and γGTP levels, and FIB-4 index are shown in Table 2. The AST, ALT, and γGTP levels significantly decreased after 6 months of tirzepatide treatment. However, the FIB-4 index remained unchanged.

**Table 2:** Changes in AST, ALT, γGTP, and FIB-4 index at 3 and 6 months after switching from dulaglutide to tirzepatide

	Baseline	3 months	6 months
AST (U/L)	30.6 ± 21.3	25.3 ± 20.8	22.2 ± 11.9 †
ALT (U/L)	34.6 ± 28.0	26.0 ± 20.2	22.6 ± 11.1 †
γGTP (mg/dL)	55.4 ± 71.2	42.3 ± 58.0	32.2 ± 32.6 †
FIB-4 index	1.49 ± 0.76	1.42 ± 0.78	1.44 ± 0.63

Data are shown as the mean ± standard deviation

*AST* aspartate aminotransferase, *ALT* alanine aminotransferase, *γGTP* γ-glutamyl transpeptidase. *FIB-4* fibrosis-4

^†^ *P* < 0.05

A total of 35% of patients experienced at least 1 treatment-emergent adverse event during the observational period. The most frequent events are gastrointestinal (diarrhea [5/40], nausea [4/40], constipation [4/40], and vomiting [2/40]), malaise (4/40), and hypoglycemia (2/40). Hypoglycemia was observed only in patients with insulin; however, severe hypoglycemia was not observed. Most of these adverse effects were transient and had resolved during the observational period. However, one patient discontinued tirzepatide due to severe nausea as described in the method section.

**Fig. 2:**
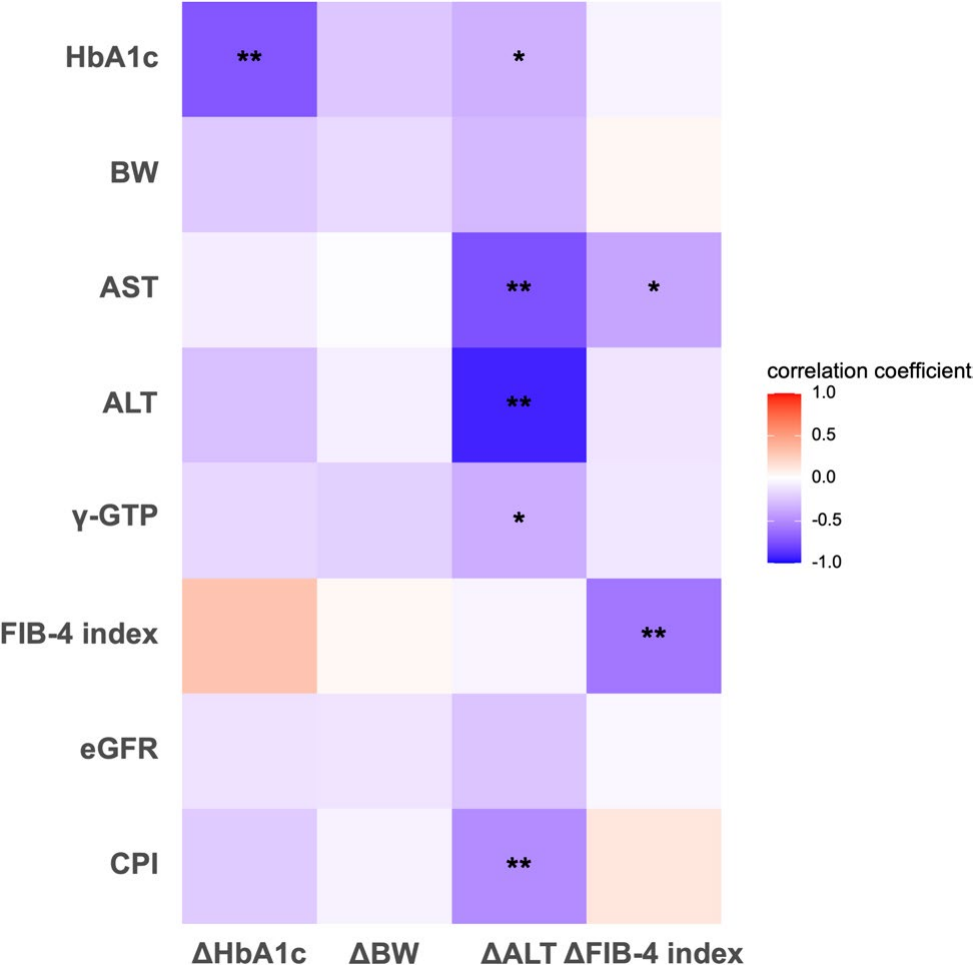
Heat map summarizing the relation between the change in glycosylated hemoglobin, body weight, alanine aminotransferase, and fibrosis-4 index and baseline characteristics. Correlation coefficients (r) are shown in the relative boxes found at the intersection between the considered variables. Positive correlation coefficients are shown using red color, whereas negative correlations are shown using blue color. Darker colors represent greater correlation, and the lighter colors represent lesser correlation. ^*^ *P* < 0.05, ^**^ *P* < 0.01

**Fig. 3:**
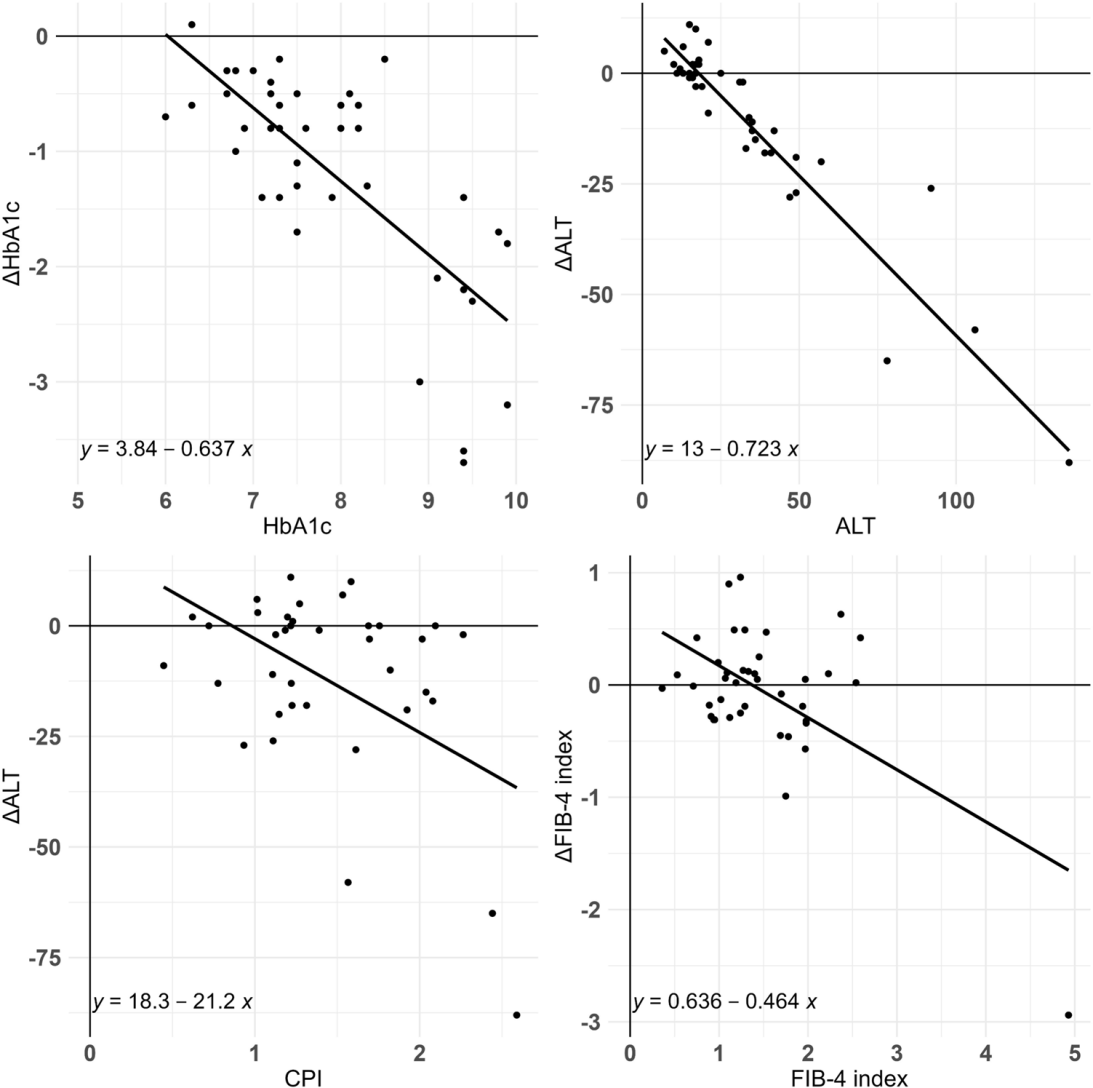
The main correlation factors between the change in each marker

The heat map displayed in Fig. 2 shows the correlation between changes in the HbA1c level, BW, ALT level, and FIB-4 index from baseline to 6 months and baseline characteristics. Darker colors represent greater correlation, and lighter colors represent lesser correlation. Among several markers shown in Fig. 2, those with significant correlations are shown in Fig. 3. The change in the HbA1c level was negatively correlated with the baseline HbA1c level but not with other factors including BMI, CPI, and liver function. In contrast, BW reduction was observed regardless of the baseline characteristics. The change in ALT level was negatively correlated with the baseline AST, ALT, and γGTP levels, and CPI. The change in the FIB-4 index was negatively correlated with the baseline AST level and FIB-4 index.

## Discussion

This study revealed that switching from dulaglutide to tirzepatide is beneficial for blood glucose level, BW, and liver function. The HbA1c reduction Decreases in blood glucose were more likely to occur in patients with higher baseline HbA1c, while decreases in body weight were seen regardless of baseline patient characteristics. Improvements in liver function, especially ALT, were more pronounced in patients with poor baseline liver function, and higher HbA1c and CPI levels.

In this study, a strong blood glucose-lowering effect was observed upon switching from dulaglutide to tirzepatide. Moreover, the blood glucose-lowering effect was stronger in patients with high HbA1c levels before the switch. However, unlike with dulaglutide [22], no correlation was observed between the change in HbA1c level and baseline BMI. Furthermore, no correlation was observed between the change in HbA1c level and baseline CPI. A sub-group analysis of SURPASS J-mono study, in which the effects of tirzepatide (5 mg, 10 mg, and 15 mg/week) and dulaglutide (0.75 mg/week) were compared for 52 weeks, showed that both dulaglutide and tirzepatide reduce postprandial glucose levels [23]. However, the mechanism of these two agents on postprandial glucose levels was different. Dulaglutide affected postprandial glucose levels mainly by enhancing early-phase insulin secretion. On the other hand, tirzepatide reduced postprandial blood glucose levels by suppressing postprandial glucagon secretion without postprandial insulin secretion [23]. Furthermore, tirzepatide increases glucose uptake in the skeletal muscle and adipose tissue compared with semaglutide independent of BW reduction in mice [24]. These findings could explain the lack of correlation between the change in HbA1c levels and CPI in this study.

Despite the strong blood glucose-lowering effect of tirzepatide, no patient experienced severe hypoglycemia during the observational period in this study. Although glycemic control is beneficial in patients with diabetes, severe hypoglycemia can increase the risk of cardiovascular events and mortality [25, 26], progression of dementia [27], and fractures [28]. Among the patients in our study, 55% of patients had agents that can induce hypoglycemia, such as insulin, sulfonylureas, and glinide. As tirzepatide has a strong blood glucose-lowering effect, appropriately evaluating the risk of hypoglycemia and considering a reduction of the dose of antidiabetic agents may be necessary.

In our study, a mean BW reduction of 3.6 kg was observed after switching from dulaglutide to tirzepatide. Excessive BW is also associated with cardiovascular complications and all-cause mortality [29]. Therefore, switching from dulaglutide to tirzepatide may be beneficial for obese patients. The SURPASS J-mono study revealed a mean BW reduction of 5.8 kg after 52 weeks in patients receiving tirzepatide 5 mg/week [20]. Among the 3.6 kg reduction of BW, approximately 80% of BW reduction was observed in the first 3 months after the switching, and another 20% was observed in the next 3 months. Although we cannot give a clear reason for the reduced weight loss effect in the second half of our study, there are several possibilities. First, In the SURPASS J-mono trial [20], two-thirds of the total BW reduction in patients receiving tirzepatide 5 mg/week was observed in the first 12 weeks. However, in the tirzepatide 10 mg and 15 mg/week groups, BW reduction was observed for a longer duration. Thus, the small BW reduction in the last 3 months of our study may be attributed to the doses of tirzepatide. Second, the impact of tirzepatide on muscle mass should be considered. Previous reports showed that the blockade of glucagon action in mice increases skeletal muscle [30]. Clinical report showed that tirzepatide increases muscle mass through the suppression of postprandial glucagon secretion [31]. The attenuated weight loss effect in the second half of treatment may have been masked by an increase in muscle mass. However, we did not evaluate body composition in this study.

In SURMOUNT-4 randomized Clinical Trial [32], in which BW changes in 52-week after 36-week of tirzepatide lead-in period was compared between two group: continued tirzepatide group and placebo group. BW changes in continued tirzepatide group and placebo group were -5.5% and + 14.0%, respectively after 20.9% of BW reduction in the lead-in period. Based on these results, it may be advisable to continue the administration of tirzepatide even if BW reduction slows down with long-term treatment. However, in our study, BW reduction occurred in all patients regardless of baseline characteristics. Therefore, it might be necessary to monitor muscle mass adequately and take precautions to avoid sarcopenia in patients without obesity after the initiation of tirzepatide.

Sugimoto et, al. reported that fat accumulation in the liver is the main cause of insulin resistance in Japanese patients with and without obesity [10]. Therefore, improvement of fatty liver is important for the treatment of diabetes. Promrat et al. reported that a 7% reduction in BW after 52 weeks of lifestyle intervention had improved steatosis, lobular inflammation, ballooning injury, and non-alcoholic steatohepatitis (NASH) histological activity scores on liver biopsy in patients with NASH [33]. Another study showed that BW reduction > 10% after lifestyle changes for 52 weeks resulted in regression of fibrosis on liver biopsy in 45% of patients, in addition to improvement in liver inflammatory marker levels [34]. However, achieving a BW reduction of 7–10% through lifestyle interventions is not easy. Several anti-diabetic agents have beneficial effects on fatty liver in patients with non-alcoholic fatty liver disease (NAFLD) and diabetes. A meta-analysis of randomized trials showed that pioglitazone improved liver fibrosis, ballooning degeneration, lobular inflammation, and steatosis [35]. Takeshita et al. reported that fibrosis scores and the histological variables, including steatosis, hepatocellular ballooning, and lobular inflammation evaluated using liver biopsy, improved after 48 weeks of tofogliflozin use [36].

In addition to several studies reporting the effects of GLP-1 analogs on AST and ALT, the beneficial effect of subcutaneous semaglutide injection therapy on liver tissue has also been reported. Liver biopsy after 72 weeks of subcutaneous semaglutide use showed 36–59% and 43% in NASH resolution and improvement in the fibrosis stage, respectively on liver biopsy [37]. There are several mechanisms of GLP-1 analogs for the improvement of liver function and liver histopathology. Improvement of insulin signals [38] and endoplasmic reticulum stress [39], promote lipolysis and fatty liver acid oxidation through family with sequence similarity 3 member A overexpression [40], the reduction in steatosis via farnesoid X receptor [41], and lowering liver inflammation via liver X receptor activation [41] were reported. In addition to these direct mechanisms of GLP-1 analogs on hepatocytes, GLP-1 analogs have been shown to significantly reduce serum C-reactive protein, interleukin-6, and tumor necrosis factor-α [42–44], which is involved in liver inflammation, development of hepatocellular carcinoma, and the progression of NASH. However, the clinical data about the effect of tirzepatide on NAFLD are unavailable.

In this study, liver function, evaluated based on AST, ALT, and γGTP levels improved. Therefore, switching from dulaglutide to tirzepatide could improve liver inflammation in patients with fatty liver and diabetes, even in the short term. In the research on mice [45], tirzepatide could improve liver steatosis and inflammation by several mechanisms than vehicle, GLP-1 analog, and GIP analog. Tirzepatide lowers the expression of genes involved in fatty acid (FA) uptake, de novo FA synthesis, and sterol excretion in the liver while increasing the expression of genes involved in de novo cholesterol synthesis and bile acid synthesis. Moreover, tirzepatide was reported to suppress inflammation in the liver by lowering the expression of CC-motif chemokine ligand 2 in the liver. The results of this study in mice corroborate our findings. However, their study [45] indicated that the expression of α-smooth muscle actin, which is expressed when resident hepatic stellate cells transform into myofibroblasts upon sensing liver injury and is involved in hepatic fibrogenesis, was lower in the livers of mice treated with tirzepatide compared to vehicle. However, the improvement in the FIB-4 index was not observed in our study. In a previous study on the effects of other classes of anti-diabetic agents, improvement in liver fibrosis was obtained at least 48 weeks of use of each agent [35–37]. Therefore, long-term observations may be required to evaluate the effect of tirzepatide on liver fibrosis.

Previous reports showed that HbA1c [46] and insulin resistance [47] are reported to be strongly associated with NAFLD. The insulin-resistance state promotes a relentless, chronic excess offer of free fatty acid as the main source of daily energy for the liver and muscle. Furthermore, Impaired cellular glucose uptake response to insulin, plus an excess influx glucose uptake response increased lipolysis of white adipose tissue, compounded by increased rates of hepatic denovo lipogenesis, leads to hepatic steatosis [48]. These findings could support the correlation between improvement in ALT and CPI in our research.

Our study had several limitations. The first limitation was the small sample size and retrospective design. Furthermore, the outcomes of our study did not include cardiovascular outcomes which is one of the most important outcomes of patients with diabetes. Therefore, prospective randomized trial with large sample size which also include cardiovascular outcomes should be conducted. Second, the changes in BW have been evaluated, but no evaluation of body composition was available in this study. Moreover, liver function was evaluated based on blood test results and not by liver biopsy. Thus, future research should include evaluations including data about body composition and liver biopsy. Third, the only pretreatment GLP-1 analog was dulaglutide. The effects of GLP-1 analogs on blood glucose and BW reduction vary between agents [49]. Dulaglutide has milder BW-decreasing effects than several other GLP-1 analogs [49]. Thus, our clinical data should be adapted to other GLP-1 analogs. As a fourth limitation, this study was conducted exclusively on Japanese participants. GLP-1 analogs achieve strong blood glucose-lowering effects and improvement of cardiovascular outcomes in Asians compared with non-Asians [50, 51]. Thus, it is not certain that our data apply to all races.

## Conclusion

Switching from dulaglutide to tirzepatide has beneficial effects on blood glucose levels, BW, and liver function in Japanese patients with type 2 diabetes.

## Data Availability

Data are available from the corresponding author on reasonable request.

## Declarations

### Ethnic approval

This study was approved by the ethics committee at the medical corporation Asanogawa (No:243, approval date:01/02/2024) with a waiver of consent obtained from the committee. Participants were recruited by the opt-out method, as this study was a retrospective observation study.

## Abbreviations

*ADA*: American Diabetes Association
*BW*: Body weight
*DPP-4*: Dipeptidyl peptidase-4
*GLP-1*: Glucagon-like peptide-1
*BMI*: Body mass index
*HbA1c*: Glycosylate hemoglobin
*eGFR*: Estimated glomerular filtration rate
*FPG*: Fasting plasma glucose
*AST*: Aspartate aminotransferase
*ALT*: Alanine aminotransferase
*γGTP*: γ-Glutamyl transpeptidase
*Cr*: Creatinine
*FIB-4*: Fibrosis 4
*CPI*: C-peptide index
*SD*: Standard deviation
*NASH*: Non-alcoholic steatohepatitis
*NAFLD*: Non-alcoholic fatty liver disease
*FA*: Fatty acid
